# Annual cycle of downward particle fluxes on each side of the Gakkel Ridge in the central Arctic Ocean

**DOI:** 10.1098/rsta.2019.0368

**Published:** 2020-08-31

**Authors:** Eva-Maria Nöthig, Catherine Lalande, Kirsten Fahl, Katja Metfies, Ian Salter, Eduard Bauerfeind

**Affiliations:** 1 Polar Biological Oceanography, Alfred Wegener Institute Helmholtz Centre for Polar and Marine Research (AWI), Am Handelshafen 12, Bremerhaven, Bremen 27570, Germany; 2 Amundsen Science, Pavillon Alexandre-Vachon, Université Laval, Québec, Québec, Canada G1 V 0A6; 3 Faroe Marine Research Institute, Tørshaven, Faroe Islands

**Keywords:** sediment traps, vertical particle flux, central Arctic Ocean, Gakkel Ridge

## Abstract

Two mooring arrays carrying sediment traps were deployed from September 2011 to August 2012 at ∼83°N on each side of the Gakkel Ridge in the Nansen and Amundsen Basins to measure downward particle flux below the euphotic zone (approx. 250 m) and approximately 150 m above seafloor at approximately 3500 and 4000 m depth, respectively. In a region that still experiences nearly complete ice cover throughout the year, export fluxes of total particulate matter (TPM), particulate organic carbon (POC), particulate nitrogen (PN), biogenic matter, lithogenic matter, biogenic particulate silica (bPSi), calcium carbonate (CaCO_3_), protists and biomarkers only slightly decreased with depth. Seasonal variations of particulate matter fluxes were similar on both sides of the Gakkel Ridge. Somewhat higher export rates in the Amundsen Basin and differences in the composition of the sinking TPM and bPSi on each side of the Gakkel Ridge probably reflected the influence of the Lena River/Transpolar Drift in the Amundsen Basin and the influence of Atlantic water in the Nansen Basin. Low variations in particle export with depth revealed a limited influence of lateral advection in the deep barren Eurasian Basin.

This article is part of the theme issue ‘The changing Arctic Ocean: consequences for biological communities, biogeochemical processes and ecosystem functioning’.

## 1. Background

Remarkable changes have been observed in the last decades in the central Arctic Ocean (CAO), including a large decrease in sea ice extent and sea ice thickness. Two extreme minima have been observed in September 2007 and 2012 and severe changes are expected to occur much earlier than previously predicted [1,2]. These changes could have a serious impact on the marine biota in the Arctic, and possible consequences for marine ecosystem services, carbon export and sequestration are currently under debate [3].

Only very few particle flux studies have been carried out so far in the entire CAO, most of them late in the productive season when access by ship is possible. Short-term deployments of ice-anchored sediment traps from late July to September showed low POC, Chl *a* and bPSi fluxes over the deep Arctic basins [4]. Spatial variability in sediment trap-derived POC fluxes concurred with the spatial variability of ^234^Th-derived POC fluxes previously measured in the CAO [5]. From observations of consistent low carbon flux in the upper surface waters despite thinner sea ice cover and more incoming light during late summer, Lalande *et al*. [4] argued that nitrate supply contributed to higher under-ice fluxes. However, Olli *et al*. [6] proposed that the low under-ice POC fluxes during the peak of the productive season resulted from the excess grazing pressure of the large copepods expatriated into the CAO. Nevertheless, under-ice blooms are occurring in some regions of the Arctic Ocean [7,8 and literature cited therein]. However, algal carbon export occurs through the export of ice algal aggregates under extensive ice cover in the CAO, mainly of *Melosira arctica* mats and some other ice-associated diatoms [9–11]. Recently, the export of an under-ice *Phaeocystis* bloom due to a ballasting effect by gypsum crystals has been reported, leading to a possible massive carbon drawdown of a pelagic bloom [12].

Whereas vertical particle flux studies have been carried out only sporadically at very few spots in the CAO, yearly sediment trap deployments for biological and biogeochemical studies have been conducted since 1999 at the long-term observatory HAUSGARTEN in Fram Strait, the deep gateway of the North Atlantic water masses to the CAO. This observatory provides much information regarding the Arctic environment and the possible effects of climate change. Fifteen years of results [13] have shown that phytoplankton and zooplankton species and particulate matter composition exported below the euphotic zone changed [14,15] with warmer Atlantic water entering the Fram Strait via the West Spitsbergen Current [16]. Among changes observed, carbonate flux increased and the populations of amphipods and pteropods changed from a dominance of cold-water species to a dominance of boreal species [17,18]. An ongoing ‘Atlantification’ of the CAO could lead to similar warming effects in the future.

Here, we present a unique study where data of vertical particle fluxes in the largely ice-covered deep Nansen and Amundsen Basins are shown. Particle flux was obtained with two mooring arrays equipped with two sediment traps, each deployed from September 2011 to August 2012 at around 83°N on both sides of the Gakkel Ridge. Whereas the mooring deployed in the Nansen Basin is connected with the North Atlantic via the Fram Strait, the mooring in the Amundsen Basin is influenced by the Lena River and the Transpolar Drift (TPD). Downward particle fluxes were measured below the euphotic zone (approx. 250 m) and ∼150 m above seafloor at ∼3500 and 4000 m, respectively. Results from the two locations were used to estimate the spatial variability in the magnitude and composition of particulate matter flux and to shed light on the modification of sinking particles on their long way down to the seafloor of the deep Arctic basins. Furthermore, our study is unprecedented since we coincidently have information on the enhanced melting period of the most recent sea ice minimum in 2012. This flux pattern will serve as a baseline and provide clues on expected changes within the next decades when the Arctic sea ice will completely melt during summer.

## 2. Methods

### (a) Sediment trap deployment

**Figure 1.**
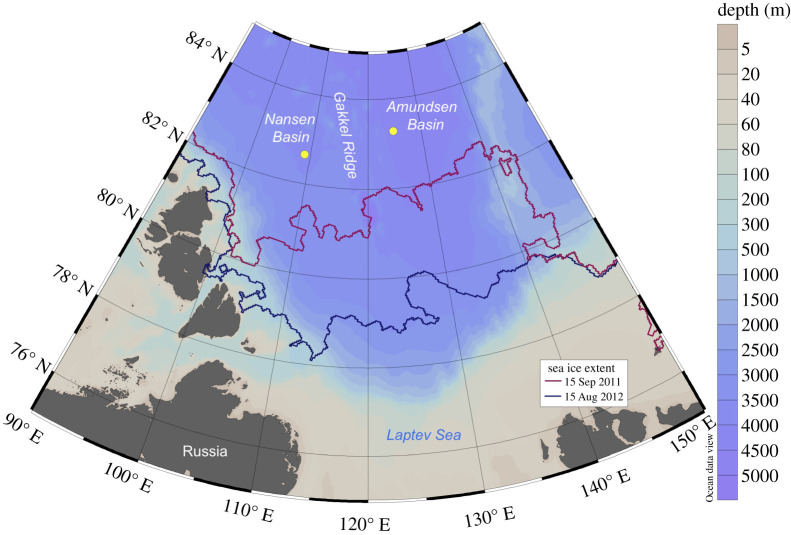
Mooring sites in the Nansen and Amundsen Basins close to the Gakkel Ridge with an indication of sea ice extent during September 2011 and August 2012. (Online version in colour.)

Sinking particles were collected with four modified cone-shaped automatic Kiel sediment traps (K/MT, sampling area 0.5 m^2^), each equipped with 20 collector cups [19]. Two bottom-tethered moorings were deployed and recovered on board RV *Polarstern* during the ARK-XXVI/3 expedition in September 2011 and the ARK-XXVII/3 expedition in August 2012. The two moorings were positioned at around 83°N on both sides of the Gakkel Ridge, one in the Amundsen Basin and one in the Nansen Basin, and the sediment traps on the moorings were deployed at approximately 250 and 150 m above seafloor (figure 1 and table 1). The traps were programmed to collect at intervals ranging from 15 days to one month during winter months. The collector cups were filled with filtered and sterilized North Sea water spiked with NaCl to adjust the salinity to 40 psu and poisoned with mercury chloride (0.14% final solution).

**Table 1.** Details of sediment trap moorings close to the Gakkel Ridge (figure 1 and electronic supplementary material, table S1).

		position				
trap name	mooring ID ARK-XXVI/3	N	E	trap depth (m)	sampling period	water depth (m)
Nansen/upper trap	Gakkel N1-1	82° 38.74'	109° 1.228'	285	15 Sep 2011–15 Aug 2012	3600
Nansen/deep trap	Gakkel N1-1	82° 38.74'	109° 1.228'	3465	15 Sep 2011–15 Aug 2012	3600
Amundsen/upper trap	Gakkel A1-1	83° 16.23'	124° 46.050'	240	15 Sep 2011–15 Aug 2012	4240
Amundsen/deep trap	Gakkel A1-1	83° 16.23'	124° 46.050'	4090	15 Sep 2011–15 Aug 2012	4240

After retrieval of the moorings, the sediment trap samples were stored refrigerated until further processing in the laboratory of the Alfred Wegener Institute Helmholtz Centre for Polar and Marine Research in Bremerhaven, Germany. In the laboratory, swimmers were removed from samples with forceps and rinsed to retrieve all particles using a dissecting microscope. The remaining particles were split into subsamples for measurements of total particulate matter (TPM), calcium carbonate (CaCO_3_), particulate organic carbon (POC), particulate nitrogen (PN), biogenic particulate silica (bPSi; bPSi values were corrected for the dissolved Si fraction measured in the supernatants of the samples), source-related biomarkers (the sea ice proxy IP_25_, the marine open water or fluvial biomarker brassicasterol, and the terrigenous biomarkers campesterol and β-sitosterol; [20] for a most recent review) and biodiversity of protists (Nansen Basin only).

### (b) Laboratory analyses

Subsamples for TPM measurements were filtered onto pre-weighed GF/F filter (nominal pore size: 0.7 µm; 25 mm ø), then rinsed with distilled water to remove salt, dried at 60°C, and weighed on a microbalance. Subsamples for CaCO_3_ measurements were also filtered onto another pre-weighed GF/F filter (nominal pore size: 0.7 µm; 25 mm ø) and treated like the TPM filters; in addition filters were soaked in 0.1N HCl to remove inorganic CaCO_3_, rinsed with Milli-Q water, and dried at 60°C, and weighed another time on a microbalance, with the carbonate fraction representing the difference between the TPM and dissolved CaCO_3_. Subsamples for POC and PN measurements were filtered onto GF/F filters (nominal pore size: 0.7 µm; 25 mm ø, pre-combusted at 500°C for 4 h), then soaked in 0.1N HCl for removal of inorganic carbon and dried at 60°C. POC and PN measurements were conducted on a Carlo Erba CHN elemental analyser and should be considered as minimum values as they were not corrected for dissolution of organic material in the collecting cups. Subsamples for bPSi measurements were filtered on cellulose acetate filters (pore size: 0.8 µm, 25 mm ø), processed using the wet-alkaline method (pre-treated for 12 h at 85°C in an oven), and extracted for 2 h at 85°C in a shaking water bath. The fraction of biogenic matter in the TPM was calculated from 2 × POC + CaCO_3_ + Opal (Opal = 2.1 × bPSi), with the lithogenic fraction representing the difference between the TPM and the biogenic matter [21].

Subsamples for biomarker analyses of brassicasterol (24-methylcholesta-5, 22E-dien-3β-ol), campesterol (24-methylcholest-5-en-3β-ol), β-sitosterol (24-ethylcholest-5-en-3β-ol) and IP_25_ were extracted with dichloromethane/methanol (1:1, v/v) and dichloromethane using separating funnels. For quantification of the lipid compounds, the internal standards 7-hexylnonadecane, C_36_ *n*-alkane and androstanol were added prior to further analytical steps. The different compounds (IP_25_ and sterols) were separated via open column chromatography (SiO_2_) using *n*-hexane (for the hydrocarbons) and ethyl acetate/*n*-hexane (20:80 v/v for sterols) as eluent. The individual sterols were silylated with 500 µl BSTFA (bis-trimethylsilyl-trifluoroacet-amide) at 60°C for 2 h. After extraction with hexane, analyses were carried out by gas chromatography-mass spectrometry (GC–MS) using an Agilent 6850 GC (30 m DB-1 MS column, 0.25 mm inner diameter, 0.25 µm film thickness) coupled to an Agilent 5975 C VL mass selective detector. Helium was used as the carrier gas. Individual compound identification was based on comparisons of their retention times with those of reference compounds and on comparisons of their mass spectra with published data [22–24]. IP_25_ was quantified using its molecular ion *m/z* 350 in relation to the abundant fragment ion *m/z* 266 of 7-hexylnonadecane and by means of an external calibration curve (*R*^2^ = 0.9989) to balance the different responses of the used ions (for further details, see [25]). Brassicasterol, campesterol and ß-sitosterol were quantified as trimethylsilyl ethers using the molecular ions *m/z* 470, *m/z* 500, *m/z* 472, and *m/z* 486, respectively, in relation to the molecular ion *m/z* 348 of androstanol.

Annual fluxes were calculated by adding the values for each sampling period multiplied by the number of days sampled. For periods not sampled (mid to end August in Nansen Basin) mid to end September 2012 for both regions, the average fluxes of August 2012 and September 2011 have been estimated.

Subsamples (total 13, table 3) were used for biodiversity analyses with Illumina Sequencing (18S meta-barcoding analysis). A fragment of the 18S rDNA containing the hypervariable V4 region was amplified with the primer set 528iF (5′-GCGGTAATTCCAGCTCC-3′) (after [26]) and 938iR (5′-GGCAAATGCTTTCGC-3′). The library preparation and sequencing of the DNA fragments were carried out with the MiSeq Reagent Kit V3 (2 × 300 bp) according to the manufacturer's protocol (Illumina, USA). Processing and DNA isolation of sediment trap samples for 18S meta-barcoding was performed as previously described [27]. Raw sequences generated in this study have been deposited at the European Nucleotide Archive (ENA) with the accession number PRJEB38972. Subsequent sequence analyses involved trimming of raw reads, chimera detection, clustering of Operational Taxonomic Units (OTUs) using a QIIME workflow with a similarity threshold of 98%, and taxonomic classification of OTUs using UCLUST as described previously [28]. In meta-barcoding studies, the term OTU classifies a group of closely related individuals reflected by gene sequence similarities [29]. Normalization, analyses and visualization of the sequence data were carried out in R (R Development Core Team, 2008). The sequence dataset was normalized using the rarefy function from the Vegan package and OTUs with sequence abundances less than 0.05% per sample were excluded from the analyses.

### (c) Remote sensing

Daily averaged sea ice concentrations were retrieved at a 12.5 km resolution from the CERSAT service of the French Research Institute for Exploitation of the Sea. Snow depth on top of sea ice was retrieved at a 25 km resolution derived from the Scanning Multichannel Microwave Radiometer and the Special Sensor Microwave/Imager of the National Aeronautics Space Agency. Apparent outliers and flagged data were removed. Daily sea ice concentration and snow depth were averaged for a delimited region (1° latitude × 1° longitude) above each mooring.

## 3. Results

### (a) Sea ice

**Figure 2.**
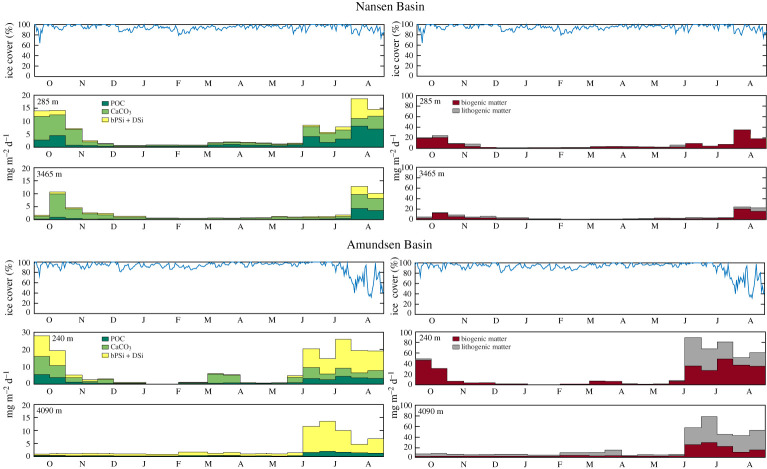
Vertical flux patterns (mg m^−2^ d^−1^) of particulate matter, left panels: POC, CaCO_3_, particulate biogenic silica (bPSi + DSi; here including the dissolved supernatant DSi); right panels: biogenic and lithogenic (a sum equal to TPM) at the two mooring sites in the Nansen (upper panels) and Amundsen (lower panels) Basins close to the Gakkel Ridge. An indication of sea ice cover during September 2011 until August 2012 is given on top of each panel. (Note the difference in *y*-axis for the upper Nansen and Amundsen traps.) (Online version in colour.)

Sea ice concentrations were typically greater than 80% all year round in the Nansen Basin, with an episodic reduction to 60–70% in September 2011 (figure 2). A similar pattern was observed in the Amundsen Basin, although a significant reduction in sea ice to below 50% occurred during July and August 2012.

### (b) Export fluxes

**Figure 3.**
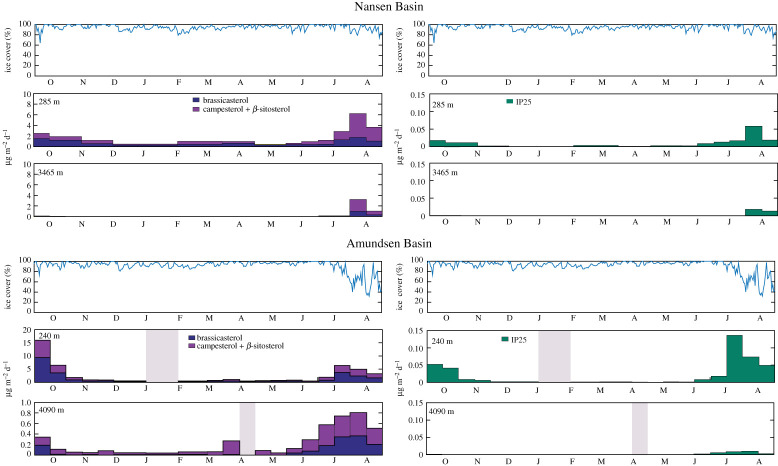
Vertical flux patterns (µg m^−2^ d^−1^) of particulate matter, left panels: brassicasterol (24-methylcholesta-5, 22E-dien-3β-ol), campesterol (24-methylcholest-5-en-3β-ol) plus β-sitosterol (24-ethylcholest-5-en-3β-ol), and right panels: IP_25_ at the two mooring sites in the Nansen (upper panels) and Amundsen (lower panels) Basins close to the Gakkel Ridge with an indication of sea ice and snow cover during September 2011 until August 2012 (% ice concentration like in figure 2, on top of each panel). (Note the difference in *y*-axis for brassicasterol, campesterol and β-sitosterol in the Nansen and Amundsen traps.) (Online version in colour.)

Generally, similar seasonal patterns of downward flux of POC and biogenic matter were observed on both sides of the Gakkel Ridge for POC and biogenic matter (figures 2 and 3; table 2). However, annual total flux (TPM, g m^−2^ y^−1^) was about three times higher in the Amundsen Basin caused by almost an order of magnitude higher flux of lithogenic material and about two times higher flux of biogenic silica (table 2). At both sites, considerable fluxes were observed from mid-September to mid-November 2011 and from June 2012 until the end of the investigation in August 2012. Peak fluxes ceased at the end of November 2011, followed by a period of low (less than 2 mg m^2^ d^−1^) residual flux for six months in the upper traps. In the deep traps, low fluxes were observed too a similar period (figures 2 and 3). Carbonate fluxes (CaCO_3_) were similar at both sampling sites but very low carbonate fluxes were recorded in the deep Amundsen trap. POC fluxes were only slightly higher in the Nansen Basin than in the Amundsen Basin. POC fluxes were greater than >5 mg C m^−2^ d^−1^ in July and August 2012 in the upper Nansen trap. The timing of enhanced POC export varied among sites and depth. During July and August 2012, POC fluxes were more or less equal in the upper and lower traps. In September/October 2011, an increase in POC fluxes was not recorded in the deep traps. What is remarkable is the very high C/N ratios (electronic supplementary material, table S1) between 15.5 and 34.8 during mid-June to mid-August 2012 in the upper Nansen trap. While high C/N ratios (above 10) were also found in the deep Nansen trap in almost all samples except for May and June 2012, in the Amundsen Basin traps C/N ratios were in most cases around 10. Export fluxes of diatom-specific bPSi remained less than 1 mg m^−2^ d^−1^ during most of the sampling period, while bPSi fluxes up to 15 mg m^−2^ d^−1^ were observed from September to October 2011 and from July to August 2012 in the Nansen Basin, and from June to August 2012 in the Amundsen Basin.

**Table 2.** Annual fluxes of particulate matter (g m^−2^ y^−1^) and biomarker (μg m^−2^ y^−1^) on both sides of the Gakkel Ridge sampled in the Nansen and Amundsen Basins.

	depth	TPM	POC	PN	C/N	bPSi + Dsi	CaCO_3_	biogenic	lithogenic	brassicasterol	campesterol + *β*-sitosterol	IP25
sediment trap	(m)	(g m^−2^ y^−1^)	(g m^−2^ y^−1^)	(g m^−2^ y^−1^)	(atomic)	(g m^−2^ y^−1^)	(g m^−2^ y^−1^)	(g m^−2^ y^−1^)	(g m^−2^ y^−1^)	(μg m^−2^ y^−1^)	(μg m^−2^ y^−1^)	(μg m^−2^ y^−1^)
Nansen Basin	285	3.258	0.802	0.051	15.7	0.371	0.938	3.135	0.123	10.46	13.71	0.16
Nansen Basin	3465	2.432	0.276	0.015	18.2	0.224	0.668	1.538	0.894	1.39	3.15	0.03
Amundsen Basin	240	8.985	0.644	0.069	9.3	1.658	1.077	5.551	3.434	27.79	23.25	0.44
Amundsen Basin	4090	7.274	0.254	0.023	11.0	0.961	0.014	2.34	4.934	1.57	3.11	0.04

**Table 3.** Species composition (biodiversity analyses with Illumina Sequencing, 18S meta-barcoding analysis) of dominating protist groups (share in %) in trap material of the upper and deep Nansen Basins during autumn 2011 and spring/summer 2012.

Nansen Basin	N-1	N-2	N-15	N-16	N-17	N-18	N-19	N-1	N-2	N-17	N-18	N-19	N-20
	285 m	285 m	285 m	285 m	285 m	285 m	285 m	3465 m	3465 m	3465 m	3465 m	3465 m	3465 m
*Month*	*Sep 11*	*Oct 11*	*June 12*	*June 12*	*July 12*	*July 12*	*Aug 12*	*Sep 11*	*Oct 11*	*July 12*	*July 12*	*Aug 12*	*Aug 12*
*Taxonomics entities (%)*													
putative picoeukaryotes	63	64	56	41	57	21	31	27	27	23	27	23	16
dinophytes	10	4	17	34	13	12	19	12	6	6	3	6	4
diatoms	0	0	4	3	1	13	3	1	2	27	35	19	21
labyrinthulomycetes	0	0	0	0	0	0	1	14	19	15	12	19	23

Source-related biomarker export fluxes, such as the marine open water or fluvial biomarker brassicasterol, were high in July and August 2012 and from September to October the year before in the upper and deep traps at both trap locations. Furthermore, brassicasterol fluxes were five times higher in the Amundsen than in the Nansen Basin from September to October. During July and August, the fluxes were nearly the same at both locations (figure 3). The same applies to the seasonal fluxes of the terrigenous sterols, i.e. the sum of campesterol and β-sitosterol. The annual fluxes of all sterols (table 2) were higher in the Amundsen Basin than in the Nansen Basin. These fluxes of brassicasterol and the terrigenous sterols were 27.79 µg m^−2^ y^−1^ and 23.25 µg m^−2^ y^−1^, respectively, in the upper trap of the Amundsen Basin and 10.46 µg m^−2^ y^−1^ and 13.71 µg m^−2^ y^−1^, respectively, in the upper trap of the Nansen Basin. The flux values in both deep traps decreased by a factor of 5–20. IP_25_ showed considerable flux rates in the upper traps during September/October 2011 and again from June to August 2012 with fairly high peaks in mid-July in the upper Nansen trap (0.06 µg m^−2^ y^−1^) and from mid-July to mid-August in the upper Amundsen trap (0.14 µg m^−2^ y^−1^), respectively. The IP_25_ flux over the Amundsen Basin was two times higher than over the Nansen Basin. The annual IP_25_ fluxes were 0.44 µg m^−2^ y^−1^ and 0.16 µg m^−2^ y^−1^ in the upper trap from both basins, respectively. The flux values in both deep traps decreased, as with the sterols, by a factor of around 10. IP_25_ was almost completely absent in both deep traps between October 2011 and May/June 2012 but was present with minor flux rates until the end of the sampling period.

Illumina sequencing of 13 samples collected from the periods of highest flux in September in the Nansen Basin generated 1 807 563 high-quality reads of the 18S rDNA V4 region. Post normalization to 106 305 sequences per sample and exclusion of OTUs with overall sequence abundance less than 0.05% the remaining sequences clustered into 627 different OTUs representing all major taxonomic groups previously found in meta-barcoding studies of marine eukaryotic microbial communities in the Arctic Ocean [26,30]. Putative picoeukaryotic and dinoflagellate OTUs contributed more than 50% of the sequences obtained from the upper trap samples, while OTUs assigned to diatoms and labyrinthulomycetes (parasites/decomposer associated with diatoms) contributed less than 5% of sequences in these samples. In the lower trap samples, the contribution of putative picoeukaryotic and dinoflagellate OTUs decreased to approximately 30%, while the contribution of OTUs assigned to diatoms and labyrinthulomycetes increased to more than 40% in most of the samples (table 3).

## 4. Discussion

Continuous sampling of export fluxes of particulate matter and biomarkers from September 2011 to August 2012 in the deep Nansen and Amundsen Basins revealed low particle fluxes, similar to the very low fluxes obtained in the Canada Basin from 2004 to 2011 [31]. Fluxes in the deep Arctic basins are much lower than average fluxes obtained in other ocean basins at depths ranging between 2000 and 4000 m ([31] and citations therein). Export fluxes were nearly negligible during the polar night under compact ice cover and increased on both sides of the Gakkel Ridge during summer. While during summertime the increase in export fluxes occurred under reduced sea ice cover during July and August in the Amundsen Basin, a similar increase was recorded over the Nansen Basin even though sea ice cover remained compact during summer. These differences in sea ice concentrations in both areas are in accordance with the three times higher fluxes of the strict sea ice-associated proxy IP_25_ in the Amundsen Basin (for review, see [20]). Higher annual fluxes of all measured parameters in the Amundsen Basin than in the Nansen Basin, except POC, possibly reflect enhanced primary production and subsequent export due to the enhanced melt, along with a larger release of ice-rafted detritus. The high C/N ratios observed in the Nansen Basin point to differences in remineralization and sinking patterns in both basins. Here, we assume higher release of nitrogen from the large ice algal aggregates that also sink fast to the deep sea. The fluxes at shallow depth from October to November are not effectively transported to depth when compared to those fluxes from June to August. This is especially true for POC and more evident in the Amundsen Basin than the Nansen Basin, suggesting that transfer efficiency is better early in the bloom export cycle. It is also of note that CaCO_3_ fluxes in the Amundsen Basin are almost completely lost, whereas there is a better transfer in the Nansen Basin. A decrease in POC:CaCO_3_ ratios from 0.85 to 0.41 in the Nansen Basin, compared to a dramatic increase from 0.59 to 18 in the Amundsen Basin, displays a large difference between the sites that likely has an important implication for effective carbon sequestration. Olli *et al*. [6] proposed that the low under-ice POC fluxes resulted from excess grazing pressure of the large copepods expatriated into the CAO which could also be an explanation for the very low biogenic fluxes from the upper trap to the deep trap in the Amundsen Basin during autumn in 2011.

Except for higher lithogenic matter fluxes observed near the seafloor, export fluxes of particulate matter decreased with depth in both the Amundsen and Nansen Basins, indicating the limited influence of lateral advection on export fluxes in the deep Eurasian Basin. This stands in strong contrast to observations in the eastern Fram Strait where particulate matter fluxes strongly increased with depth [32] but is in accordance with observations of other deep ice-covered basins [31,33]. Most parameters show a relatively high decline during descent, with biomarker fluxes displaying the most abrupt decrease in the Amundsen Basin, indicating a possible biogeochemical degradation with increasing water depth. Here, the terrigenous sterols are the most stable compounds [25]. The abrupt decrease can also be reinforced by an incorporation of organic carbon including the sea ice proxy IP_25_ and the marine open water/fluvial brassicasterol as well as the terrigenous sterols [25] into the marine food web [34,35]. Although grazing may be important in reducing the export fluxes [6], only few zooplankton fecal pellets were observed in the trap samples, suggesting a strong degradation of organic matter in Arctic surface waters. POC and PN fluxes also showed a strong decrease with depth but were less extreme.

A shift in biodiversity was observed in the deep trap of the Nansen Basin. The proportion of labyrinthulomycetes and diatoms increased with depth in the Nansen Basin, indicating a close connection between sea ice algal assemblages [30] and the deep sea organisms [36]. They may have been transported via the sinking of the larger aggregates of the ice-associated *M. arctica*. Microscopic analyses of the upper sediment trap samples in both the Nansen and Amundsen Basins indicated the export of single cells of the ice-associated and IP_25_-producing *Haslea* spp. and of short chains and resting spores of *M. arctica* [11]. Further microscopic analyses of the deep trap samples revealed larger and stickier aggregates of *M. arctica*. The export of sea ice algae was more pronounced in the Nansen Basin than in the Amundsen Basin, where fewer *M. arctica* cells but more pelagic diatoms such as *Chaetoceros* spp. and *Thalassiosira* spp. were found in the upper and deep trap material. The larger contribution of pelagic diatoms to the flux in the Amundsen Basin is most probably due to the influence of sea ice incorporated diatoms that were transported from the Laptev Sea shelf and the Lena River outflow within the Transpolar Drift (TPD) regime. This is also in agreement with the higher fluxes of the terrigenous sterols, which derived from vascular land plants of the Siberian hinterland [23,24]. Recently, it was reported [37] that more of the now thinner sea ice is assumed to melt earlier before reaching the Fram Strait via the TPD so that pelagic diatoms may have been transported this way to the deep Amundsen trap samples. Higher biogenic silica and lithogenic fluxes and enhanced ice melt in the Amundsen Basin than in the Nansen Basin support this hypothesis.

Nevertheless, the changing composition of the sinking particles with depth revealed a high sedimentation rate during ice melt from June/July to September in the high Arctic transporting the bulk of the released ice algae aggregates with moderate loss of matter on the way down to roughly 4000 m. Lalande *et al.* [11] and previous investigations, e.g. [9], reported that exclusively ice-associated algae like *Nitzschia frigida* and *M. arctica* contributed to a large proportion of the annual organic matter fluxes in the Eurasian Arctic. Benthic megafauna and sediment bacteria of the deep Arctic basins may benefit from ice algae deposition [10], maybe even more in the future.

## Supplementary Material

Annual cycle of downward particle fluxes on each side of the Gakkel Ridge in the Central Arctic Ocean

## Data accessibility

All data will be available in the public repository PANGAEA without restriction under doi:10.1594/PANGAEA.915057 and doi:10.1594/PANGAEA.915058.
