# Supplementary material for: Annual cycle of downward particle fluxes on each side of the Gakkel Ridge in the central Arctic Ocean
Source: Philos Trans A Math Phys Eng Sci. 2020 Aug 31;378(2181):20190368. doi: 10.1098/rsta.2019.0368 (PMC7481669; doi:10.1098/rsta.2019.0368)
Supplement: Annual cycle of downward particle fluxes on each side of the Gakkel Ridge in the Central Arctic Ocean [file rsta20190368supp1.docx]

**Annual cycle of downward particle fluxes on each side of the Gakkel Ridge in the Central Arctic Ocean**

Nöthig, Eva-Maria^1^; Lalande, Catherine^2^; Fahl, Kirsten^1^; Metfies, Katja^1^; Salter, Ian^1,3^; Bauerfeind, Eduard^1^

^1^Alfred Wegener Institute Helmholtz Centre for Polar and Marine Research (AWI) - Am Handelshafen 12, D-27570 Bremerhaven, Germany

^2^Amundsen Science, Pavillon Alexandre-Vachon, Université Laval, Québec, Québec, G1V 0A6, Canada

^3^Faroe Marine Research Insitute, Tørshaven, Faroe Islands

ORCID: EMN, 0000-0002-7527-7827; CL, 0000-0002-1416-7288; KF, 0000-0001-9317-4656; KM, 0000-0003-3073- 8033; IS, 0000-0002-4513-0314; EB, 0000-0002-6945-8218

**Keywords:** sediment traps, vertical particle flux, Central Arctic Ocean, Gakkel Ridge

**Supplementary Figures & Tables**

Table S1: C/N ratios in the different traps samples; bold C/N > 20.

| **Nansen 285 m** | **Sample Glass** | **Duration** | **Sampling time** | **C/N** | **Amundsen 245 m** | **Sample Glass** | **Duration** | **Sampling time** | **C/N** |
| --- | --- | --- | --- | --- | --- | --- | --- | --- | --- |
| Month |  | Days |  |  | Month |  | Days |  |  |
| *September* | 1 | 15 | 15.09.11-30.09.11 | 10.2 | *September* | 1 | 15 | 15.09.11-30.09.11 | 10.1 |
| *October* | 2 | 15 | 30.09.11-15.10.11 | 9.7 | *October* | 2 | 15 | 30.09.11-15.10.11 | 8.9 |
| *October* | 3 | 16 | 15.10.11-31.10.11 | 9.1 | *October* | 3 | 16 | 15.10.11-31.10.11 | 7.5 |
| *November* | 4 | 15 | 31.10.11-15.11.11 | 7.7 | *November* | 4 | 15 | 31.10.11-15.11.11 | 8.0 |
| *November* | 5 | 15 | 15.11.11-30.11.11 | 7.4 | *November* | 5 | 15 | 15.11.11-30.11.11 | 7.3 |
| *December* | 6 | 31 | 30.11.11-31.12.11 | 8.2 | *December* | 6 | 31 | 30.11.11-31.12.11 | 7.3 |
| *January* | 7 | 31 | 31.12.11-31.01.12 | 7.7 | *January* | 7 | 31 | 31.12.11-31.01.12 | no data |
| *February* | 8 | 29 | 31.01.12-29.02.12 | 7.6 | *February* | 8 | 29 | 31.01.12-29.02.12 | 7.9 |
| *March* | 9 | 15 | 29.12.12-15.03.12 | 8.0 | *March* | 9 | 15 | 29.12.12-15.03.12 | 8.3 |
| *March* | 10 | 16 | 15.03.12-31.03.12 | 9.3 | *March* | 10 | 16 | 15.03.12-31.03.12 | 8.2 |
| *Apil* | 11 | 15 | 31.03.12-15.04.12 | 8,7 | *Apil* | 11 | 15 | 31.03.12-15.04.12 | 6.1 |
| *April* | 12 | 15 | 15.04.12-30.04.12 | 7.3 | *April* | 12 | 15 | 15.04.12-30.04.12 | 5.6 |
| *May* | 13 | 15 | 30.04.12-15.05.12 | 8.4 | *May* | 13 | 15 | 30.04.12-15.05.12 | 8.2 |
| *May* | 14 | 15 | 15.05.12-30.05.12 | 6.8 | *May* | 14 | 15 | 15.05.12-30.05.12 | 7.5 |
| *June* | 15 | 16 | 30.05.12-15.0612 | **31.1** | *June* | 15 | 16 | 30.05.12-15.0612 | 8.6 |
| *June* | 16 | 15 | 15.06.12-30.06.12 | **24.5** | *June* | 16 | 15 | 15.06.12-30.06.12 | 11.1 |
| *July* | 17 | 15 | 30.06.12-15.07.12 | **15.5** | *July* | 17 | 15 | 30.06.12-15.07.12 | 10.8 |
| *July* | 18 | 16 | 15.07.12-31.07.12 | **34.8** | *July* | 18 | 16 | 15.07.12-31.07.12 | 11.4 |
| *August* | 19 | 15 | 31.07.12-15.08.12 | **23,0** | *August* | 19 | 15 | 31.07.12-15.08.12 | 9.2 |
|  |  |  |  |  |  |  |  |  |  |
| **Nansen 3465 m** | **Sample Glass** | **Duration** | **Sampling time** | **C/N** | **Amundsen 4090 m** | **Sample Glass** | **Duration** | **Sampling time** | **C/N** |
| Month |  | Days |  |  | Month |  | Days |  |  |
| *September* | 1 | 15 | 15.09.11-30.09.11 | 15.9 | *September* | 1 | 15 | 15.09.11-30.09.11 | 14.0 |
| *October* | 2 | 15 | 30.09.11-15.10.11 | 29.8 | *October* | 2 | 15 | 30.09.11-15.10.11 | 10.9 |
| *October* | 3 | 16 | 15.10.11-31.10.11 | 22.2 | *October* | 3 | 16 | 15.10.11-31.10.11 | 13,5 |
| *November* | 4 | 15 | 31.10.11-15.11.11 | 12.9 | *November* | 4 | 15 | 31.10.11-15.11.11 | 13.4 |
| *November* | 5 | 15 | 15.11.11-30.11.11 | 14.3 | *November* | 5 | 15 | 15.11.11-30.11.11 | 11.6 |
| *December* | 6 | 31 | 30.11.11-31.12.11 | 13.0 | *December* | 6 | 31 | 30.11.11-31.12.11 | 10.7 |
| *January* | 7 | 31 | 31.12.11-31.01.12 | 15.2 | *January* | 7 | 31 | 31.12.11-31.01.12 | 11.5 |
| *February* | 8 | 29 | 31.01.12-29.02.12 | 14.2 | *February* | 8 | 29 | 31.01.12-29.02.12 | 10.9 |
| *March* | 9 | 15 | 29.12.12-15.03.12 | 14.3 | *March* | 9 | 15 | 29.12.12-15.03.12 | 10.4 |
| *March* | 10 | 16 | 15.03.12-31.03.12 | 17.6 | *March* | 10 | 16 | 15.03.12-31.03.12 | 12.5 |
| *Apil* | 11 | 15 | 31.03.12-15.04.12 | 15.6 | *Apil* | 11 | 15 | 31.03.12-15.04.12 | 11.2 |
| *April* | 12 | 15 | 15.04.12-30.04.12 | 12.4 | *April* | 12 | 15 | 15.04.12-30.04.12 | 10.8 |
| *May* | 13 | 15 | 30.04.12-15.05.12 | 7.7 | *May* | 13 | 15 | 30.04.12-15.05.12 | 10.4 |
| *May* | 14 | 15 | 15.05.12-30.05.12 | 6.9 | *May* | 14 | 15 | 15.05.12-30.05.12 | 8.5 |
| *June* | 15 | 16 | 30.05.12-15.0612 | 7.9 | *June* | 15 | 16 | 30.05.12-15.0612 | 10.9 |
| *June* | 16 | 15 | 15.06.12-30.06.12 | 7.9 | *June* | 16 | 15 | 15.06.12-30.06.12 | 9.8 |
| *July* | 17 | 15 | 30.06.12-15.07.12 | 11.1 | *July* | 17 | 15 | 30.06.12-15.07.12 | 13.1 |
| *July* | 18 | 16 | 15.07.12-31.07.12 | 17.5 | *July* | 18 | 16 | 15.07.12-31.07.12 | 10.1 |
| *August* | 19 | 15 | 31.07.12-15.08.12 | **22.0** | *August* | 19 | 15 | 31.07.12-15.08.12 | 9.3 |
|  |  |  |  |  |  |  |  |  |  |
